# A Tunable Sponge-like Lipophilic Gel with Branched Poly(2-propyl aspartamide) Crosslinkers for Enhanced VOC Absorption

**DOI:** 10.3390/gels11040286

**Published:** 2025-04-13

**Authors:** Sunggyu Shin, Naseul Jung, Hyewon Jeong, Eunjin Heo, Kyungsuk Cho, Jaehyun Jeong

**Affiliations:** 1 Department of Chemical Engineering, Soongsil University, Seoul 06978, Republic of Korea; whitegd45@soongsil.ac.kr (S.S.); naseul94@naver.com (N.J.); hw1205d@naver.com (H.J.); ejhur97@naver.com (E.H.); 2 Department of Environmental Science and Engineering, Ewha Womans University, Seoul 03760, Republic of Korea

**Keywords:** sponge-like lipophilic gel, VOCs absorption, grafted crosslinker, porous structure

## Abstract

In this study, we present a sponge-like lipophilic gel crosslinked with a branched crosslinker as an absorbent for VOC removal. The gel was synthesized by crosslinking the monomer 3-(trimethoxysilyl)propyl methacrylate (TMSPMA) with the branched crosslinker poly(2-propyl aspartamide) grafted methacrylate (PPA-g-MA). The grafted crosslinker, PPA-g-MA, was prepared by introducing acrylate groups as crosslinking moieties to the poly(succinimide) precursor for poly(2-propyl aspartamide) (PPA), which serves as a hydrophobic backbone. Lipophilic gels were synthesized with varying TMSPMA monomer concentrations and freeze-dried to form a porous structure. To evaluate VOC absorption, the toluene removal efficiency of the sponge-like lipophilic gel was tested in a continuous gas flow system. As a result, the optimal TMSPMA monomer content for maximizing toluene removal efficiency was determined. This result suggests that while an increase in silicon content generally enhances VOC removal efficiency, the porous structure of sponge-like lipophilic gels plays a more crucial role in absorption capacity. The collapse of the porous structure, caused by excessive silicon content making the material more rubber-like, explains why there exists an optimal monomer content for effective VOC absorption. Overall, these findings provide valuable insights for developing high-performance VOC absorbents.

## 1. Introduction

Volatile organic compounds (VOCs) have received growing attention as significant air pollutants due to their high volatility, reactivity, and toxicity to both humans and the environment [1,2,3,4]. In recent years, VOCs have been recognized as major factors in air pollution, as they pose serious health risks, including acute and chronic respiratory effects, neurotoxicity, lung cancer, and irritation of the eyes and throat [5,6,7]. Additionally, VOCs contribute to severe environmental issues such as global warming, stratospheric ozone depletion, fine particulate matter (PM) formation, and photochemical smog. These compounds are emitted not only from industrial processes that use organic solvents but also from everyday sources such as printing, dry cleaning, and food processing industries, making their mitigation essential [8,9].

Various approaches have been investigated to reduce and eliminate VOC emissions, including adsorption, condensation, bioreactors, and membrane separation [10,11,12,13,14]. However, bioreactors and membrane separation processes are mainly applicable in large-scale industrial complexes that generate substantial VOC emissions [15,16,17]. For smaller-scale industries and indoor environments, adsorption-based filtration systems using activated carbon have been widely utilized [18,19]. Nevertheless, activated carbon suffers from drawbacks such as limited VOC adsorption capacity, frequent replacement cycles, and a rapid decline in adsorption performance over time. More recently, liquid-phase absorbents, such as silicone oils, have been explored as an alternative for VOC removal due to their high affinity for VOC molecules [20,21,22]. However, these liquid absorbents present challenges, including limited contact area with VOCs and difficulties in regeneration through absorption–desorption cycling. Despite numerous studies on VOC removal, current methods remain constrained by their inherent limitations. Previous research has demonstrated the effectiveness of lipophilic gels in VOC removal, showing high swelling ratios in various VOC solutions and excellent reusability [23,24]. Furthermore, the incorporation of branched crosslinkers previously in studies of ours optimized absorption capacity while maintaining mechanical strength [24].

**Figure 1:**
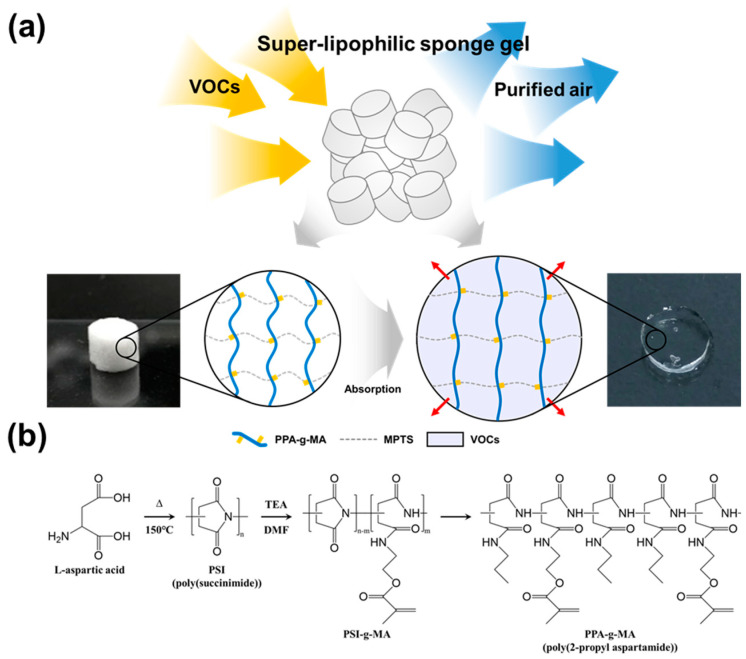
Schematic of Sponge-like Lipophilic Gel for VOCs Absorption. (**a**) The dried sponge-like lipophilic gel, with a porous structure, absorbs VOCs, which accumulate and liquefy, causing the gel to swell. (**b**) A grafted poly(amino acid) crosslinker was synthesized and introduced as a hydrophobic crosslinker into the lipophilic gel.

Therefore, in this study, we propose a novel VOC removal strategy using a sponge-like lipophilic gel synthesized with a branched hydrophobic crosslinker (PPA-g-MA), which forms a porous structure via freeze-drying to simultaneously enhance gas–gel contact (adsorption) and internal VOC uptake (absorption) (Figure 1). The lipophilic gel was fabricated by crosslinking 3-(trimethoxysilyl)propyl methacrylate (TMSPMA) with the grafted crosslinker PPA-g-MA, which was synthesized by introducing methacrylate groups to the hydrophobic poly(2-propyl aspartamide) backbone. The porous structure was achieved by freeze-drying, and the toluene removal efficiency was evaluated in a continuous gas flow system. Through varying the TMSPMA monomer content, we determined the optimal composition that balances silicon content and pore retention. Moreover, the sponge-like morphology of the lipophilic gel not only increased the contact area with VOC gases, enhancing absorption efficiency, but also enabled repeated reuse through absorption–drying cycles, thereby addressing limitations seen in conventional liquid absorbents. This study not only proposes a new method for VOC removal using a sponge-like lipophilic gel but also provides insights into strategies for enhancing absorbent performance. The findings are expected to contribute to advancements in VOC mitigation technologies and their practical application in various industrial sectors that emit VOCs.

## 2. Results and Discussion

### 2.1. Synthesis of Poly(2-propyl aspartamide) Grafted Methacrylate

**Figure 2:**
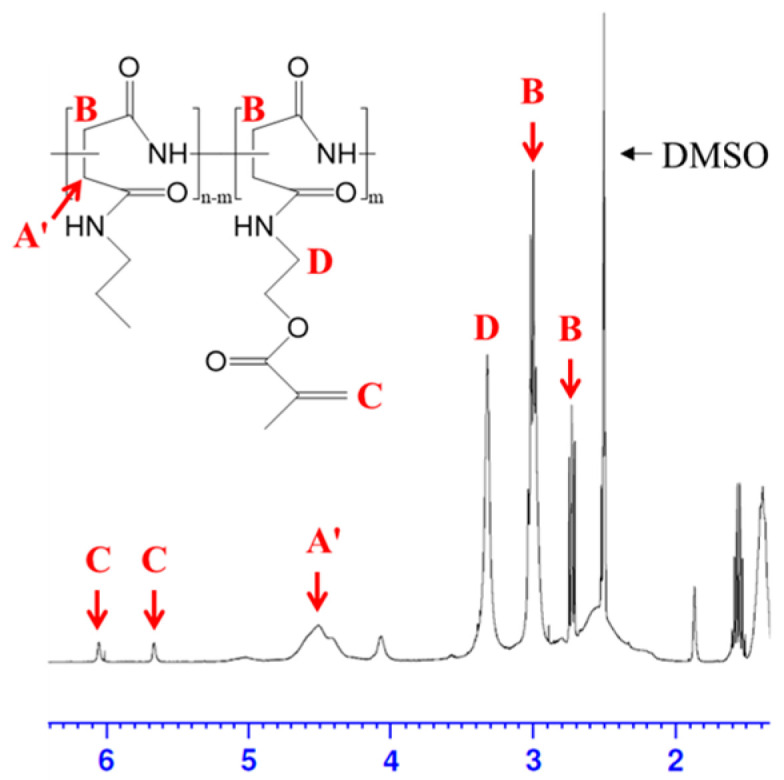
^1^H-NMR spectrum and structure of PPA-g-MA.

To fabricate the sponge-like lipophilic gel, we synthesized a grafted crosslinker, poly(2-propyl aspartamide) grafted methacrylate (PPA-g-MA), which was incorporated into the gel for enhanced VOC absorption. The synthesis was initiated via preparation of the precursor polymer, poly(succinimide) (PSI, Mw: 19,000 g/mol), through the polymerization of L-aspartic acid using phosphoric acid as a catalyst. Methacrylate groups were introduced into the polymer at grafting degrees of 10%, 20%, and 30%, corresponding to mol% per PSI unit. The degree of substitution (DS) was defined as the molar ratio of amine-containing monomer units to total succinimide units. The DS value was determined based on the integration area from the ^1^H-NMR spectra. The molecular structures of PSI, PSI-g-MA, and PPA-g-MA were successfully confirmed through ^1^H-NMR analysis (Figure 2). Characteristic peaks for PSI units appeared at 2.7 ppm and 3.2 ppm (methylene), 5.3 ppm (methine), and for the introduced acrylate groups at 5.6 ppm and 6.0 ppm (methylene) and 1.87 ppm (methyl). The actual DS values were calculated using the characteristic peaks of PSI (-CH) and methacrylate (-CH_3_), yielding final DS values of 6%, 13%, and 17% as shown in Table 1 [24].

(1) $${DS}_{MA}(\%)=\frac{The\;integral\;of\;the\;pick\;1.8\;\sim \;1.9ppm/3}{The\;integral\;of\;the\;pick\;5.1\;\sim \;5.5ppm}\times 100$$

### 2.2. Optimization of Swelling and Mechanical Properties of Lipophilic Gel

**Figure 3:**
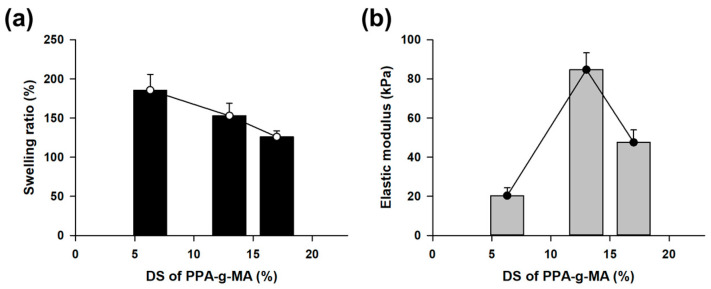
Analysis of the physical properties of the lipophilic gel based on the DS of the PPA-g-MA crosslinker. (**a**) The swelling ratio decreased as the DS increased while maintaining a constant acrylate concentration. (**b**) The elastic modulus exhibited an increasing trend followed by a decline, reaching its highest value at DS 13%.

The lipophilic gel was prepared through thermal-radical polymerization using 2.0 M 3-(trimethoxysilyl)propyl methacrylate (TMSPMA) and PPA-g-MA crosslinkers with varying degrees of substitution (DS). In this polymerization, the acrylate concentration of PPA-g-MA was fixed at 0.04 M across all samples. The gel was analyzed to examine the correlation between the swelling ratio and elastic modulus. Typically, gels exhibit an inverse relationship between the swelling ratio and elastic modulus [25]. However, this lipophilic gel demonstrated a unique behavior where swelling and mechanical strength were independently controlled by fixing the acrylate number of the crosslinker at different DS. As the DS of PPA-g-MA increased, the swelling ratio decreased from 185% to 126% (Figure 3a). In contrast, the elastic modulus reached its highest value of 84 kPa at a DS of 13% (Figure 3b). This result indicates that both the swelling ratio and the elastic modulus decreased in tandem as the DS increased from 13% to 17%. Based on these findings, we selected PPA-g-MA with a DS of 13% to synthesize a lipophilic gel that maintains an adequate swelling ratio while offering superior mechanical strength.

### 2.3. Toluene Absorption Behavior of Sponge-Like Lipophilic Gel in a Continuous-Gas Flow Column System

**Figure 4:**
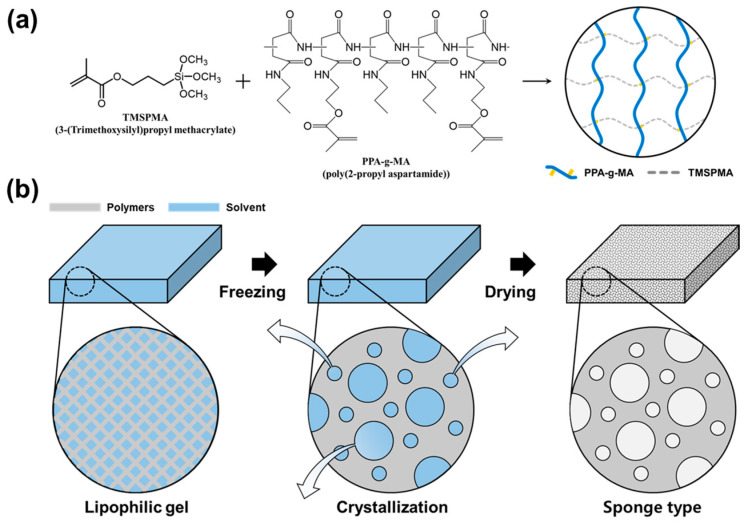
Sponge-like lipophilic gel was prepared by polymerizing TMSPMA and PPA-g-MA, followed by freeze-drying. (**a**) First, TMSPMA and PPA-g-MA were polymerized to synthesize the lipophilic gel. (**b**) The synthesized gel was rapidly frozen and freeze-dried to remove the internal solvent, resulting in a porous sponge-like structure.

A lipophilic gel was synthesized by polymerizing TMSPMA and PPA-g-MA (Figure 4a). Subsequently, freeze-drying was employed to remove the solvent from the gel, thereby creating a porous structure (Figure 4b). Unlike our previous work using PSI-g-MA for liquid-phase VOC absorption in non-porous gel matrices, this study introduces a more hydrophobic crosslinker (PPA-g-MA) and employs freeze-drying to fabricate a sponge-like structure optimized for gas-phase VOC removal. Effective VOC removal relies on the initial contact between the absorbent material and VOC molecules. In this study, the introduction of a porous structure increased the surface area available for absorption, enhancing the initial adsorption efficiency. As a result, the gel rapidly absorbed airborne VOCs while maintaining consistent absorption performance. Furthermore, the structural stability of the material was preserved, ensuring its durability after VOC absorption.

**Figure 5:**
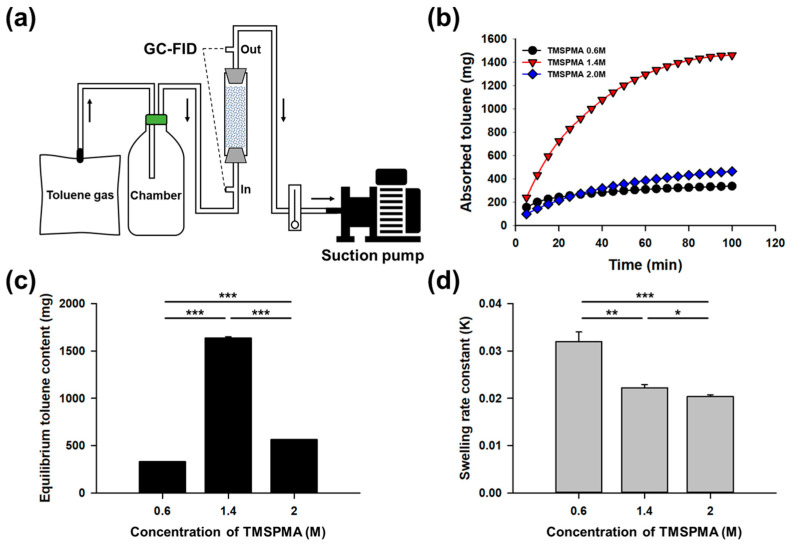
(**a**) A continuous gas flow system was designed, where sponge-like lipophilic gel was packed into a column and continuously exposed to toluene gas. The difference in toluene concentration between the inlet and outlet of the column was analyzed using GC. (**b**) Toluene absorption was low at TMSPMA concentrations of 0.6 M and 2.0 M, whereas the 1.4 M concentration exhibited superior absorption performance. (**c**) At equilibrium, the highest amount of toluene was retained in the column packed with the 1.4 M TMSPMA gel. (**d**) The swelling rate constant was highest at 0.6 M, while the values for 1.4 M and 2.0 M were similar. (* *p* < 0.1, ** *p* < 0.05, *** *p* < 0.01; statistical significance determined by one-way ANOVA followed by Tukey’s test.).

To evaluate the removal efficiency of gaseous toluene, a continuous gas flow system was designed, where toluene was continuously introduced into a column packed with sponge-like lipophilic gel (Figure 5a). The columns were filled with lipophilic gels synthesized with TMSPMA concentrations of 0.6 M, 1.4 M, and 2.0 M. The amount of toluene absorbed by the sponge-like lipophilic gel within the column was calculated based on the difference between the inlet and outlet toluene concentrations. The time-dependent toluene absorption in the column is presented in Figure 5b. Among the tested samples, the sponge-like lipophilic gel synthesized with 1.4 M TMSPMA exhibited the most superior performance. In this system, the 1.4 M TMSPMA gel demonstrated a rapid initial absorption rate (Figure 5b) and the highest equilibrium toluene content within the column, reaching 1634 mg (Figure 5c). In contrast, the sponge-like lipophilic gel synthesized with the highest silicon content (2.0 M TMSPMA) exhibited significantly lower performance, with a slower initial absorption rate and a relatively low equilibrium toluene content of 562 mg. Despite its high silicon content and inherent potential for toluene absorption, the 2.0 M TMSPMA gel failed to achieve optimal absorption efficiency, suggesting that excessive silicon content may negatively impact the gel’s absorption capability.

### 2.4. Swelling Kinetics of Sponge-Like Lipophilic Gels

The swelling rate of the sponge-like lipophilic gel was calculated based on the change in toluene content within the column. Figure 5d presents the swelling rate constants of the sponge-like lipophilic gels. The swelling rate constant is an indicator of how quickly an equilibrium is reached [26,27]. The TMSPMA 0.6 M sponge-like lipophilic gel contained the least amount of TMSPMA among the tested gels, resulting in a lower capacity for toluene absorption. Consequently, this gel reached equilibrium the fastest and exhibited the highest swelling rate constant of 0.032, indicating that it rapidly attained its maximum absorption capacity. The TMSPMA 1.4 M sponge-like lipophilic gel, which had the highest toluene content at equilibrium, showed swelling rate constants of 0.022 and 0.020, similar to the TMSPMA 2.0 M gel. These values indicate that although both 1.4 M and 2.0 M gels had similar swelling rates, the 1.4 M gel demonstrated a more favorable balance between swelling speed and capacity. This suggests that an optimal TMSPMA content can provide both sufficient porosity and appropriate crosslinking density, enabling not only rapid toluene uptake but also high equilibrium absorption. In contrast, excessive crosslinking in the 2.0 M gel likely reduced pore accessibility, limiting its kinetic performance despite similar rate constants. These results suggest that the TMSPMA 1.4 M sponge-like lipophilic gel absorbed toluene gas more rapidly and sustained absorption more effectively compared to the TMSPMA 2.0 M gel. Since silicon-based materials generally serve as VOC-absorbing media, an increase in silicon content typically enhances toluene absorption. However, the results of this study reveal an opposite trend in gaseous toluene absorption. This discrepancy is likely due to structural changes within the sponge-like lipophilic gel as the silicon content increases. Higher silicon content makes the gel structure more rubber-like, reducing its porosity and thereby decreasing the surface area available for initial toluene gas contact. In contrast, the TMSPMA 0.6 M sponge-like lipophilic gel retains its porous structure, but its lower TMSPMA content leads to rapid saturation, limiting its overall toluene absorption capacity.

**Figure 6:**
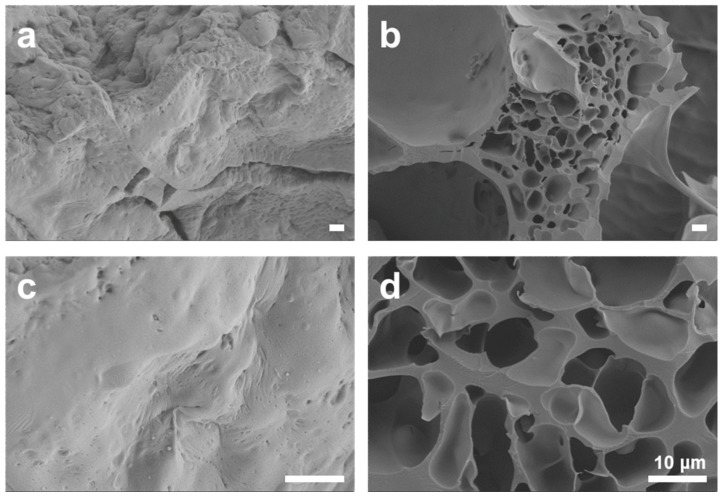
The internal structure of the sponge-like lipophilic gel was examined using scanning electron microscopy (SEM). (**a**,**b**) The TMSPMA 2.0 M gel lost its porous structure due to the rubberization of silicon. (**c**,**d**) In contrast, the TMSPMA 0.6 M gel maintained its porous structure. All scale bars represent 10 μm.

To investigate the porosity of the sponge-like lipophilic gel, scanning electron microscopy (SEM) analysis was performed. As shown in Figure 6a,b, the TMSPMA 2.0 M sponge-like lipophilic gel exhibited a lack of porosity and a rubberized structure, visually demonstrating the reduced contact area with gaseous toluene. In contrast, the TMSPMA 0.6 M sponge-like lipophilic gel successfully maintained a porous structure (Figure 6c,d). This indicates that a sponge-like porous structure enhances the contact area, facilitating faster VOC gas absorption. Despite its higher potential absorption capacity, the structural limitations prevented the gel from achieving optimal absorption performance. These findings highlight that, in VOC absorption materials, not only the content of key components but also structural factors play a crucial role in the adsorption and absorption processes. Overall, the sponge-like lipophilic gel developed in this study effectively increased the contact surface area through its porous structure, successfully enhancing its intrinsic absorption capacity, making it a promising VOC absorbent.

## 3. Conclusions

This study demonstrates the potential of sponge-like lipophilic gels as effective VOC absorbents and presents an advanced strategy for enhancing absorption capacity through a combination of silicone content and porous structure. The lipophilic gel was synthesized via the polymerization of a grafted crosslinker (PPA-g-MA) and the silicone monomer TMSPMA. Among the synthesized gels, the formulation with DS 13% under identical acrylate conditions exhibited the highest elastic modulus, allowing the stable formation of a porous structure by freeze-drying. The VOC absorption capacity was evaluated by adjusting the TMSPMA concentration to 0.6 M, 1.4 M, and 2.0 M. Under a continuous gas flow system, the gel containing 1.4 M TMSPMA demonstrated the highest toluene absorption at equilibrium. This superior performance can be attributed to its porous structure, which provided an increased contact area, leading to efficient initial VOC uptake. In contrast, the 2.0 M TMSPMA gel exhibited rubber-like characteristics, preventing the formation of a porous structure and ultimately resulting in lower toluene absorption. Overall, these findings provide a new perspective on improving the performance of VOC absorbent materials, shifting the focus from simply increasing silicone content to optimizing structural properties. By achieving a balance between porosity and material composition, this study offers valuable insights into the development of next-generation VOC removal technologies. The sponge-like lipophilic gel developed here holds significant potential for various applications, including VOC capture and environmental remediation.

## 4. Materials and Methods

### 4.1. Materials

L-aspartic acid, sulfolane, phosphoric acid (85%), N,N-dimethylformamide (DMF) (99.0%), 2-aminoethyl methacrylate hydrochloride (AEMA), trimethylamine (TEA), propylamine, and dimethyl sulfoxide (DMSO) were purchased from Sigma-Aldrich (St. Louis, MO, USA). 3-(Trimethoxysilyl)propyl methacrylate (TMSPMA) and acrylamide (AAm) were also obtained from Sigma-Aldrich. Methanol, ethyl ether, and azobisisobutyronitrile (AIBN) were purchased from Samchun (Seoul, Korea).

### 4.2. Synthesis of Grafted Crosslinker PPA-g-MA

Poly(succinimide) (PSI) was synthesized via an acid-catalyzed polymerization of L-aspartic acid. L-aspartic acid (0.188 mol) was suspended in sulfolane (125 g) in the presence of phosphoric acid (9.4 mmol) and stirred at 150 °C under a nitrogen (N_2_) flow. Water generated during the reaction was continuously removed using a Dean–Stark trap and a reflux condenser. After 15 h, the reaction mixture was precipitated in excess methanol, and residual acid catalyst was removed by washing with deionized water until a pH of 7.0 was achieved [24,28]. The resulting poly(succinimide) (PSI) was dissolved in DMF, and 2-aminoethyl methacrylate hydrochloride (AEMA) and trimethylamine (TEA) were added in a molar ratio of 1:3. The reaction was carried out for 24 h under a nitrogen flow to prepare the poly(succinimide)-grafted methacrylate (PSI-g-MA). Residual byproducts (TEA-HCl) were removed using a syringe filter (0.45 μm, Corning, New York, NY, USA). The mixture was precipitated in excess ethyl ether and dried at 80 °C. Excess propylamine was then added to the PSI-g-MA dissolved in DMF, and the reaction was carried out at room temperature for 24 h to prepare the poly(2-propyl aspartamide)-grafted methacrylate (PPA-g-MA). Finally, the synthesized PPA-g-MA was precipitated in ethyl ether and dried at room temperature to yield the final product. The chemical structures of PSI, PSI-g-MA, and PPA-g-MA were confirmed *via* ¹H-NMR spectroscopy (400 MHz, AVANCE III, BRUKER, Billerica, MA, USA).

### 4.3. Preparation of Lipophilic Gel

The lipophilic gel was prepared by polymerizing the synthesized PPA-g-MA crosslinker with the silicone monomer TMSPMA. The resulting lipophilic gel was designed to evaluate the toluene absorption capacity and mechanical properties as a function of the crosslinker’s degree of substitution (DS). First, TMSPMA was added to DMSO at a concentration of 2.0 M. PPA-g-MA was added at a fixed acrylate concentration of 0.04 M for each DS. Azobisisobutyronitrile (AIBN) was used as an initiator at a 1.0% concentration to prepare the pre-gel solution. The pre-gel solution was placed between two quartz plates with a 1 mm spacer and polymerized at 70 °C for 2 h. After polymerization, the quartz plates were separated, and the gel was punched into standardized discs with a diameter of 8 mm and thickness of 1 mm using an 8 mm biopsy punch (Miltex, York, PA, USA). The final lipophilic gel was immersed in DMSO to reach full swelling, and the swollen gel was used for further property analysis.

The stiffness of the lipophilic gel was evaluated by measuring the elastic modulus (E) using a universal testing machine (UTM, DrTech, Seongnam, Republic of Korea). The lipophilic gel was punched out and fully swollen in DMSO. The gel was then compressed at a constant deformation rate of 1.0 mm/s. The elastic modulus was calculated using the following equation:

(2) $$E=\frac{\sigma \left(\varepsilon \right)}{\varepsilon }=\frac{F/A}{∆L/L_{0}}$$

where *F* is the force exerted on a gel under tension, *A* is the actual cross-sectional area, Δ*L* is the amount by which the length of the gel changes, and $L_{0}$ is the original length of the gel. The elastic modulus was calculated as the slope of the stress (*σ*) vs. the strain (*ε*) curve for the first 2~8% strain [29].

The swelling property of the gel was dependent on the stiffness and structure of the polymer. The swelling ratio (Q) of the lipophilic gel was calculated via the following equation:

(3) $$Q=\frac{W_{s}-W_{d}}{W_{d}}\times 100\left(\%\right)$$

where $W_{s}$ is the weight of the fully swelled gel and the $W_{d}$ is the weight of the dried gel. The weight of the dried gel was measured after it was freeze-dried [30].

### 4.4. Fabrication of Sponge-Like Lipophilic Gel

The sponge-like lipophilic gel was prepared by freeze-drying the lipophilic gel synthesized through the thermal polymerization reaction of TMSPMA and PPA-g-MA. For this, PPA-g-MA with a degree of substitution (DS) of 13% was used to optimize the mechanical strength and swelling ratio of the lipophilic gel. The lipophilic gel was prepared in two groups: one with TMSPMA added at a concentration of 2.0 M and the other with reduced TMSPMA content, compensated for by the addition of acrylamide (AAm). The molar ratios of TMSPMA to AAm were 7:3 and 3:7, respectively, with the monomer concentration fixed at 2.0 M. AAm; a monomer typically used in hydrogels was utilized here to lower the density of the silicon within the polymer, as it does not absorb in hydrophobic solvents. Following this, azobisisobutyronitrile (AIBN) was added at 1.0% to prepare the pre-gel solution. The pre-gel solution was transferred to a flat-bottomed reactor, purged with nitrogen gas, and heated at 70 °C for 2 h. After polymerization, the lipophilic gel was retrieved and standardized into cylindrical shapes with a diameter of 8 mm and a height of approximately 10 mm using an 8 mm biopsy punch. The gel was then immersed in DMSO for 24 h to remove any unreacted materials. To create a sponge-like porous structure, the lipophilic gel was rapidly cooled in liquid nitrogen and then freeze-dried. The gel was dried for 7 days, and the final sponge-like lipophilic gel was stored in a desiccator.

### 4.5. Application of Sponge-Like Lipophilic Gel in a Continuous Gas Flow Column System

The toluene absorption capability of the sponge-like lipophilic gel was analyzed in a continuous gas flow system. First, 5 mL of sponge-like lipophilic gel was packed into a 7 mL cylindrical column. Toluene gas, at a concentration of 40,000 ppm, was passed through the column at a flow rate of 30 mL/min using a pump (AIRWELL2010, Odor-tech, Paju, Republic of Korea), designed to flow uniformly and continuously. The concentrations of toluene entering and exiting the column were measured every 5 min using GC-FID.

The sponge-like lipophilic gels absorb toluene in the continuous gas flow system. In this system, the sponge-like lipophilic gel swelled gradually by absorbing more toluene. During absorption, the weight of toluene was measured GC-FID every 5 min. The swelling rate, $r_{s}$, was calculated using the following equation:

(4) $$r_{s}=\frac{{dW}_{t}}{dt}=K_{s}\left(W_{eq}-W_{t}\right)$$

where $K_{s}$ is the swelling rate constant, $W_{eq}$ is the weight of toluene under swelling equilibrium, and $W_{t}$ is the weight of toluene at time *t*. The swelling kinetic, $K_{s}$, was further calculated from the slope of the ln[($W_{eq}$ − $W_{0}$)/($W_{eq}$ − $W_{t}$)] versus the time curve using the following equation [29,31]:

(5) $$\ln\left[\frac{W_{eq}-W_{0}}{W_{eq}-W_{t}}\right]=K_{s}t$$

### 4.6. Statistical Analysis

Statistical significance was determined using one-way ANOVA followed by Tukey’s Multiple Comparison Test (* *p* < 0.1, ** *p* < 0.05, *** *p* < 0.01).

## Figures and Tables

**Table 1:** Characteristics of the synthesized PPA-g-MA.

Sample	Feed ^a^	DS ^b^	Number ^c^
PPA	100/0	-	-
PPA-g-MA 1	90/10	6	12
PPA-g-MA 2	80/20	13	25
PPA-g-MA 3	70/30	17	33

^a^ Feed mole ratio (mol%, succinimide unit/methacrylate). ^b^ Degree of substitution(mol%) determined based on ^1^H-NMR of graft copolymers. ^c^ Number of methacrylates per one polymer chain.

## Data Availability

Data are contained within the article.
